# Association of Test Anxiety with Temporomandibular Disorder in Health Professions Students: A Cross-Sectional Study

**DOI:** 10.1155/2020/8833804

**Published:** 2020-12-10

**Authors:** Abdulaziz Alamri, Suliman Shahin, Eman A. Bakhurji, Ahmed A. Alsulaiman, Zainah Salloot, Muhammad Nazir

**Affiliations:** Department of Preventive Dental Sciences, College of Dentistry, Imam Abdulrahman Bin Faisal University, Dammam 31441, Saudi Arabia P. O. Box 1982

## Abstract

**Objective:**

To assess the prevalence of temporomandibular disorder (TMD) and its association with text anxiety among undergraduate medical, dental, and pharmacy students in Dammam, Saudi Arabia. *Material and Methods*. This cross-sectional study included health professions students who responded to Fonseca's questionnaire and Test Anxiety Inventory by Spielberger to evaluate TMD and test anxiety, respectively. TMD score was compared in different categories of students, and bivariate and multiple logistic regression analyses evaluated the influence of test anxiety on TMD.

**Results:**

The study included 884 participants (44.8% males and 55.2% females) with a mean age of 21.46 ± 1.36 years. Regarding items of Fonseca's questionnaire, most students reported being tense/nervous (65.7%) and had headaches (57.5%). About 45.8% of the participants reported no TMD, and remaining had mild (40.4%), moderate (11.3%), and severe (2.5%) TMD. The mean TMD score was significantly higher in students with high test anxiety (25.6 ± 18.32) than those with low test anxiety (20.25 ± 16.97) (*P* < 0.001). Mean test anxiety scores significantly differed among TMD categories (*P* < 0.001) with the lowest score in the no TMD group and the highest in the moderate TMD group. Female gender (adjusted odds ratio 1.4, P 0.039) and high test anxiety (adjusted odds ratio 1.92, *P* < 0.001) were significantly associated with increased odds of having TMD.

**Conclusions:**

The study revealed a high prevalence of TMD among students. There was a significant association between test anxiety and TMD. The data obtained may guide preventive policies and program on test anxiety and TMD.

## 1. Introduction

Temporomandibular disorder is a complex group of degenerative musculoskeletal conditions usually portrayed in orofacial pain and undermined mastication, and it can affect up to 25% of young adults [1]. Although, evidence supports presence of TMD in any age group, however, most patients between the age of 20 and 50 years present with the symptoms of TMD [1, 2]. TMD can be essentially segmented to myofacial pain dysfunction disorders, internal derangements, and degenerative joint diseases [3]. TMD has diverse anatomic, physiologic, genetic, and psychological underlying factors [2]. The diagnosis of TMD chiefly relies on signs and symptoms of a patient including pain, clicking jaw sounds, and limitation of mouth opening [4]. Masticatory muscular pain and fatigue, increased teeth wear and mobility, headaches, tinnitus, and photophobia are other signs and symptoms of TMD [5]. A systematic review confirmed that TMD symptoms negatively affected oral health-related quality of life [6].

Test anxiety is defined as a “set of phenomenological, physiological, and behavioral responses that accompany concern about possible negative consequences or failure on an exam” [7]. It affected 25% to 40% of students in the United States [8]. Almost every individual in the formal education had experienced test anxiety at some point of his or her educational pathway [9]. Test anxiety has been associated with academic underachievement and it can interfere with achieving academic and, consequently, professional goals [10]. Embarrassment, fear, and feeling pressured, and unfavorable effects such as tachycardia, xerostomia, excessive perspiration, stomach pain, and frequent urination can occur in individuals affected with test anxiety [11].

In Saudi Arabia, studies reported a high prevalence of TMD and exam anxiety in health professions students [12–14]. Stress is one of the important factors in the development of TMD [3]. It is assumed that increased levels of test anxiety can affect TMD in students of health professions who routinely take a variety of examinations and assessments in their intense programs. However, the literature lacks data about the influence of test anxiety on TMD in undergraduate health professions students. Hence, this study aimed to assess the association between TMD and text anxiety among undergraduate medical, dental, and pharmacy students.

## 2. Material and Methods

### 2.1. The Study Population

This cross-sectional study conducted an empirical investigation of health professions students' responses about text anxiety and TMD. The sampling frame included all undergraduate medical, dental, and pharmacy students in Dammam, Saudi Arabia. The sample size calculation was based on the expected proportion of TMD in the population (50%), 95% confidence level, and the total width of confidence (0.06). This resulted in a sample of 1098 participants. The study participants conveniently participated in the study and data were collected in September–November 2019.

In order to survey students, structured self-administered questionnaires were distributed to male and female students in their respective colleges. The research included students from all years, and the questionnaires were administered in their classes and were collected immediately after completion. The subjects with abnormalities of teeth, jaw, and soft tissues, those with significant systemic or psychological disorders, those under the treatment of TMD, subjects having orofacial pain, and subjects taking analgesics were excluded from the study.

### 2.2. Measurement Instrument

The data were collected using a questionnaire that includes items about demographic information of study participants, and Fonseca's questionnaire and Test Anxiety Inventory by Charles D. Spielberger. Test anxiety was the exposure, and TMD was the outcome in the study. Fonseca's questionnaire was proposed by Fonseca and is commonly used to evaluate the severity of TMD [15]. The instrument has a multidimensional evaluation and is highly effective in collecting epidemiological data in a short time [16, 17]. It is composed of 10 questions each with three options such as “Yes, Sometimes, and No.” These 10 questions ask respondents about the difficulty in opening the mouth and moving jaw to the sides, pain in muscle on chewing, headache, neck stiffness, earache, noise in TMJ on mouth opening, parafunctional habits, perception about malocclusion, and nervousness. For the analysis of the responses, a value of 10 is given to Yes; 5 for Sometimes; and 0 for No. The score of the questionnaire can range from 0 to 100. The total score of each participant is calculated to classify the severity of TMD using the Fonseca index classification. Accordingly, a person with a score of 0–15 points is categorized as not having TMD, mild TMD (20–40 score), moderate TMD (45–65 score), and severe TMD (70–100 score).

Spielberger's Test Anxiety Inventory Scale consists of 20 items that is used to measure the test anxiety of college students. Each item uses a 4-point Likert type scale, and participants respond to the four options: “Almost Never, Sometimes, Often, and Almost Always” [18]. The total test anxiety score ranges from a minimum of 20 to a maximum of 80 [19]. The instrument has acceptable validity and internal reliability [20].

### 2.3. Pretesting of the Questionnaire

The study questionnaire was piloted among 30 medical, dental, and pharmacy students. Pilot testing helped ensure the readability and ease of understanding of the questions by the participants.

### 2.4. Ethics and Consent

The study was approved by the ethics committee at the College of Dentistry. Permission to conduct the survey was obtained from the administration in medical, dental, and pharmacy colleges. Before the distribution of questionnaire, the participants received instructions about the objectives and details of the study including potential risks and benefits. The participants provided their informed consent. The study was conducted in accordance with the ethical principles of the Declaration of Helsinki.

### 2.5. Statistical Analysis

The data were analysed using Statistical Package for Social Sciences Version 22.0 (IBM SPSS Statistics for Windows, Armonk, NY: IBM Corp). The questionnaires with missing or incomplete information were excluded from statistical analysis. Descriptive statistics included the frequency distribution of the questionnaire responses, means, and standard deviations of continuous variables. The chi-square test was performed to compare the TMD score in terms of gender, age, and class year. The independent *t*-test and ANOVA test were performed to compare the TMD score in different categories of students. The mean Test Anxiety Inventory scores were compared among participants with no TMD, mild TMD, moderate TMD, and severe TMD. Bivariate and multiple logistic regression analyses were performed to evaluate the association between test anxiety and TMD. Multiple logistic regression analysis was used to control for confounding variables when assessing the relationship between test anxiety and TMD. A *P* value <0.05 was considered statistically significant.

## 3. Results

The study included 884 participants (mean age of 21.46 ± 1.36 years) with a response rate of 80.5%. The majority of the participants were from dental college (46%), and most were females (55%). The mean score of TMD was 23.03 ± 17.87, and the mean score of test anxiety was 42.87 ± 10.85 (Table 1).

In Table 2, “Yes” and “Sometimes” responses about the items of Fonseca's questionnaire were combined for the ease of interpretation of results. Being tense/nervous was the most common problem reported by the participants (65.7%), and this was followed by headaches (57.5%) and neck pain or a stiff neck (39.6%). Difficulty in moving the jaw to the side was the least frequently encountered problem (12.2%).

Analysis of mean scores of TMD was performed to identify differences in different categories of students. Findings showed statistically significant (*P* 0.021) differences in the mean scores of TMD in dental (23.3 ± 18.02), medical (21.01 ± 16.39), and pharmacy students (25.66 ± 9.47). Female students (24.1 ± 17.57) demonstrated a significantly higher mean TMD score than male students (21.7 ± 18.17) (*P* 0.047). The mean TMD score was found significantly higher in students with high test anxiety (25.6 ± 18.32) compared with students with low test anxiety (20.25 ± 16.97) (*P* < 0.001) (Table 3).

Female gender (unadjusted odds ratio 1.3, *P* 0.03) and high test anxiety (unadjusted odds ratio 1.86, *P* < 0.001) were significantly associated with TMD in bivariate analysis. After adjustment for other factors in multiple logistic regression, students had 1.92 times higher odds of having TMD if they had high test anxiety (*P* < 0.001). Also, female students were 1.4 times more likely to have TMD compared to male students (P 0.039). However, the study found no significant association of GPA, parental education, and monthly family income with TMD (Table 4).

**Figure 1:**
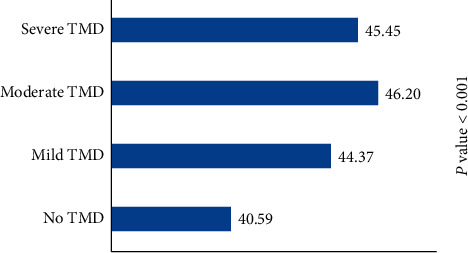
Comparison of mean test anxiety score in different categories of TMD.

After categorizing the TMD scores based on the severity of symptoms, 46% of participants had no TMD, while 40% had mild TMD, 11% had moderate TMD, and only 2.5% had severe TMD symptoms. Mean test anxiety scores significantly differed among TMD categories (*P* < 0.001) with the lowest score in students with no TMD and the highest in students with moderate TMD (Figure 1).

## 4. Discussion

The literature reports the prevalence estimates of TMD in university students based on the Fonseca questionnaire in the range of 22.6% to 68%. In our study, 54.2% of the participants had mild to severe forms of TMD which is comparable to the findings in the literature. A Brazilian study reported TMD in 53.21% of undergraduate dental students [16]. Another similar study observed TMD in 68% of university students in Brazil [21]. In Saudi Arabia, 43.48% of medical college students were found to have mild to severe TMD [22]. In Riyadh, 46.8% of male students of medicine, dentistry, pharmacy, applied medical sciences, and engineering reported TMD [12]. Another team of researchers reported that the prevalence of TMD was 53.3% in male and female undergraduate students of dentistry, medicine, and pharmacy and engineering in Madinah, Saudi Arabia [13]. TMD was observed in 42.9% of university students in Taiwan [23], and 30.6% of medical and dental students demonstrated TMD in Nepal [24]. However, in India, 22.6% of university students reported the symptoms of TMD [25]. These variations in the prevalence estimates can be related to racial differences, demographic factors in each study, and diversity in study methodology [12, 16]. Despite differences in the prevalence, mild-TMD category was the most common type of TMD in university students in most studies [12, 13, 24–26].

Stress is one of the documented causative factors of TMD in addition to trauma, occlusal discrepancies, and increased masticatory muscle tension [27]. Also, the development of TMD symptoms is related to stress, anxiety, and depression in university students [16, 26, 28]. The university students with TMD were found to have higher stress and general anxiety scores compared to those students without TMD [13]. Nomura et al. reported nervousness in 76.72% and headache in 64.66% of dental students [16]. Nervousness was also the most common problem in university students reported by Habib et al. [12] These findings are similar to our study, where 65.7% of students reported nervousness and 57.5% headaches which were the most common symptoms of TMD. Health science students are vulnerable to psychological problems such as stress, anxiety, and depression because of academic demands and challenges of providing quality treatment and care to patients [29]. This may account for a high prevalence of nervousness and headache in health professions students.

The results from the current study had revealed that students with low test anxiety experienced significantly lower TMD than those with high test anxiety. The logistic regression model showed that test anxiety contributed significantly to TMD and students with high test anxiety had 1.86 times higher odds of having TMD than those with low test anxiety. Furthermore, test anxiety was significantly associated with the severity of TMD. Similar to the role of psychological factors in the development of TMD, test anxiety is assumed to increase the risk of TMD in health sciences students [26]. This data supporting the association between test anxiety and TMD can be of great importance for the prevention and management of these conditions particularly in health professions students who are more prone to develop TMD than other students [25]. However, the association through the application of Fonseca's questionnaire and Test Anxiety Inventory should be further investigated.

Our study showed female gender predilection with TMD. The mean TMD score was significantly higher in female than in male students. Similarly, after controlling for other variables, female students were 1.4 times more likely to have TMD than male students. These results were consistent with a large body of evidence. TMD symptoms more frequently reported in female than male students in a previous study in Saudi Arabia [13]. In India, the prevalence of TMD was about twice higher in female than male university students [25]. Studies of university students in Brazil reported a significant association between female gender and TMD [16, 26]. Gender difference in physiological traits, pain threshold, muscular and connective structure, and hormonal variations can be attributed to female gender associated with TMD [21, 25, 26].

The assessment of TMD in different health professions students in the current study indicated that pharmacy students had the highest score of TMD compared with medical and dental students. This differs with the findings by Zafar et al. who found that TMD was most commonly distributed in dental (46.6%) than pharmacy (34%) and medical students (19.3%) [13]. Similar trends were observed in a recent study of Brazilian students where 75.34% of dental, 70% of pharmacy, and 64.7% of medical students had mild to severe TMD [26]. Whether certain health professions have a higher propensity for TMD is worth investigating on the basis of controlled educational factors. Particular attention should be given to the evaluation of dental/oral and systemic factors contributing to the risk of TMD in addition to the academic environment, including types of training students receive.

Although our study used a large sample of health professions students and provided reliable evidence about the prevalence and severity of TMD and its relationship with test anxiety. However, the results of this study should be interpreted considering its limitations. The study included a conveniently selected sample. The students from a public university participated in the study, thus limiting generalizability of study findings. Therefore, the results of the study should not be generalized to the students from a private institution or other geographical areas of the country. There is some possibility of over-reporting of TMD symptoms in health professions students because they may be more aware of TMD. The reasons for nonresponse included the absence of students on the day of data collection and students' refusal to participate in the study. Cross-sectional study design allows the investigation of the association between different variables, but no cause and effect inferences can be made. In the future, clinical longitudinal studies should be conducted to evaluate the role of test anxiety in the development and progression of TMD. Furthermore, acquiring test anxiety during various periods in an academic calendar might be of particular interest to evaluate the differential effect of stress periods on TMD.

## 5. Conclusions

- The symptoms of nervousness and headaches and TMD were highly prevalent among health professions students.
- The female gender was significantly associated with an increased likelihood of TMD.
- Significant differences in TMD score were observed between students with high and low test anxiety.
- Test anxiety was significantly associated with the severity of TMD. Students with high test anxiety were significantly twice more likely to have TMD than students with low test anxiety.
- Study findings may guide in developing programs and policies to prevent and control test anxiety and TMD in health professions students.

## Figures and Tables

**Table 1:** Characteristics of study participants.

Study variables	*N* (%) *N* = 884
College	
Dental college	410 (46.4)
Medical college	293 (33.1)
Pharmacy college	181 (20.5)
Gender	
Male	396 (44.8)
Female	488 (55.2)
Age	Mean ± SD 21.46 ± 1.36
Mean score of TMD	23.03 ± 17.87
Mean score of test anxiety	42.87 ± 10.85

**Table 2:** Distribution of study participants' responses to Fonseca's questionnaire.

Questions	Yes *N* (%)	Sometimes *N* (%)	Combined Yes/Sometimes *N* (%)	No *N* (%)
1. Do you have difficulty opening your mouth wide?	44 (5)	142 (16.1)	186 (21)	698 (79)
2. Do you have difficulty moving your jaw to the side?	36 (4.1)	72 (8.1)	108 (12.2)	776 (87.8)
3. Do you feel fatigue or muscle pain when you chew?	38 (4.3)	181 (20.5)	219 (24.8)	665 (75.2)
4. Do you have headaches?	138(15.6)	370 (41.9)	508 (57.5)	376 (42.5)
5. Do you have neck pain or a stiff neck?	97 (11)	253 (28.6)	350 (39.6)	534 (60.4)
6. Do you have earache or pain in that area (temporomandibular joint)?	49 (5.5)	134 (15.2)	183 (20.7)	701 (79.3)
7. Have you ever noticed any noise in your temporomandibular joint while chewing or opening your mouth?	117 (13.2)	184 (20.8)	301 (34)	583 (66)
8. Do you have any habits such as clenching or grinding your teeth?	112 (12.7)	165 (18.7)	277 (31.3)	607 (68.7)
9. Do you feel that your teeth do not come together well?	182 (20.6)	150 (17)	332 (37.6)	552 (62.4)
10. Do you consider yourself a tense (nervous) person?	214 (24.2)	367 (41.5)	581 (65.7)	303 (34.3)

**Table 3:** Comparison of TMD score in different categories of study participants.

Study variables	Mean TMD score	*P* value
College		
Dental college	23.3 ± 18.02	0.021^*∗*^
Medical college	21.01 ± 16.39	
Pharmacy college	25.66 ± 19.47	
Gender		
Male	21.7 ± 18.17	0.047^*∗*^
Female	24.1 ± 17.57	
Academic year		
Preclinical years	24.78 ± 18.87	0.080
Clinical years	22.39 ± 17.47	
Grade point average (GPA)		
Low	21.93 ± 18.13	0.146
High	23.72 ± 17.69	
Father's education level		
No/school education	22.57 ± 16.71	0.577
College/university education	23.28 ± 18.49	
Mother's education level		
No/school education	24.18 ± 18.4	0.096
College/university education	22.16 ± 17.43	
Monthly family income (*N* = 656)		
Low	23.75 ± 16.99	0.783
Middle	23.66 ± 18.28	
High	22.71 ± 17.82	
Test anxiety		
Low	20.25 ± 16.97	<0.001^*∗*^
High	25.6 ± 18.32	

**Table 4:** Association of different factors with TMD in study participants.

Study variables	Unadjusted odds ratio (95% confidence interval)	*P* value	Adjusted odds ratio (95% confidence interval)	*P* value
College	0.86 (0.65, 1.14) 1.11 (0.8, 1.54)	0.309 0.534	1 (0.73, 1.38) 1.07 (0.75, 1.54)	0.979 0.703
Dental college^*∗*^				
Medical college				
Pharmacy college				
Gender	1.34 (0.57, 0.97)	0.030	1.4 (1.02, 1.94)	0.039
Male				
Female^*∗*^				
Academic year	1.21 (0.89, 1.63)	0.224	1.33 (0.96, 1.86)	0.090
Preclinical years^*∗*^				
Clinical years				
Grade point average (GPA)	0.8 (0.61, 1.05)	0.103	0.89 (0.64, 1.23)	0.478
Low^*∗*^				
High				
Father's education level	0.9 (0.68, 1.18)	0.438	0.97 (0.71, 1.32)	0.832
No/school education				
College/university education^*∗*^				
Mother's education level	0.86 (0.66, 1.12)	0.263	0.91 (0.67, 1.22)	0.524
No/school education				
College/university education^*∗*^				
Monthly family income (*N* = 656)	1.48 (0.92, 2.38) 1.02 (0.76, 1.36)	0.101 0.893	1.45 (0.86, 2.44) 1.01 (0.74, 1.38)	0.165 0.946
Low				
Middle				
High^*∗*^				
Test anxiety	1.86 (1.42, 2.43)	<0.001	1.92 (1.46, 2.53)	<0.001
Low				
High^*∗*^				

^*∗*^Reference categories.

## Data Availability

The SPSS data file of this study is available from the corresponding author upon request.
